# MicroRNAs in Metastasis and the Tumour Microenvironment

**DOI:** 10.3390/ijms22094859

**Published:** 2021-05-04

**Authors:** Carla Solé, Charles Henderson Lawrie

**Affiliations:** 1 Molecular Oncology Group, Biodonostia Research Institute, 20014 San Sebastian, Spain; charles.lawrie@biodonostia.org; 2 IKERBASQUE, Basque Foundation for Science, 48009 Bilbao, Spain; 3 Radcliffe Department of Medicine, University of Oxford, Oxford OX4 3DU, UK

**Keywords:** tumour microenvironment, miRNAs, metastasis

## Abstract

Metastasis is the process whereby cancer cells migrate from the primary tumour site to colonise the surrounding or distant tissue or organ. Metastasis is the primary cause of cancer-related mortality and approximately half of all cancer patients present at diagnosis with some form of metastasis. Consequently, there is a clear need to better understand metastasis in order to develop new tools to combat this process. MicroRNAs (miRNAs) regulate gene expression and play an important role in cancer development and progression including in the metastatic process. Particularly important are the roles that miRNAs play in the interaction between tumour cells and non-tumoral cells of the tumour microenvironment (TME), a process mediated largely by circulating miRNAs contained primarily in extracellular vesicles (EVs). In this review, we outline the accumulating evidence for the importance of miRNAs in the communication between tumour cells and the cells of the TME in the context of the pre-metastatic and metastatic niche.

## 1. Introduction

Cancer metastasis, the spread of tumour cells from the primary tumour site, has been reported to account for approximately 67–90% of cancer-related deaths. Approximately half of all cancer patients present with metastasis at the time of diagnosis [1,2,3]. Metastasis is a multistep process, which starts when tumour cells detach from the primary tumour mass, intravasate into lymphatic and circulatory systems to become circulating tumour cells (CTCs), extravasate to leave the circulation, invade and proliferate in a new niche of a distant tissue/organ to form a new tumour [4]. The metastatic process is very inefficient since from the 0.2% of CTCs that survive their time in circulation, only those cells that are the first to reach permissive target organs and are then able to colonize those tissues can initiate metastatic tumour growth [5].

It is well-known that cancer initiation and progression, as well as metastasis, not only depends on tumour cells themselves, but also on the cells of the tumour microenvironment (TME) [6,7,8]. The major components of the TME apart from tumour cells include cancer-associated fibroblasts (CAFs), endothelial cells and immune cells, in addition to other components such as the extracellular matrix [9,10,11]. Hypoxia, cellular oxygen deprivation, is an important factor that drives many aspects of metastasis [12,13], including the initiation of the epithelial–mesenchymal transition (EMT) process that changes the phenotype of tumour cells allowing them to escape from the matrix of the primary tumour [14]. In addition to hypoxia, the interaction between tumour cells and the TME induces a wide range of biological events that are necessary for metastasis including proliferation, immunosuppression and angiogenesis [15,16,17]. Many of these processes are regulated by microRNAs (miRNAs) and are the subject of this review. In addition to the direct control of metastasis by miRNAs, it has been recently shown that they can regulate the metastatic process by acting as mediators of intercellular communication between tumour cells and cells of the TME [18,19].

MiRNAs are a class of small (19–25 nucleotides) non-coding single-strand RNAs. Since their initial discovery in *Caenorhabditis elegans* [20], miRNAs have been demonstrated to play key roles in many, if not all, physiological cellular functions by regulating target genes through primarily negative post-transcriptional regulation of gene expression [21,22,23]. A single miRNA is capable of targeting many genes and, conversely, a single gene can be targeted by many miRNAs leading to a complex regulatory network that encompasses more than 60% of human genes [24]. In addition to their importance under physiological conditions, miRNAs are ubiquitously deregulated in cancer and can act as tumour-promoting miRNAs (oncomiRNAs and metastamiRNAs) targeting messenger RNAs (mRNAs) coding for proteins that act as tumour suppressors or as tumour suppressor miRNAs targeting mRNAs coding for proteins with oncogenic properties [25].

In 2007, two separate reports released in parallel first described the association between miRNAs and metastasis. Ma et al. demonstrated that miR-10b could promote breast cancer metastasis in vitro and in vivo through targeting of the HOXD10 (Homeobox D10) gene [26], whilst Yu et al. demonstrated that let-7 can act as a metastasis suppressor miRNA through targeting of H-RAS and HMGA2 (High Mobility Group AT-Hook 2), leading to a reduction in proliferation, mammosphere formation and metastatic potential, including in breast cancer [27]. Subsequently, many miRNAs have been identified that are associated with metastasis or with associated pathways, such as migration and invasion [28].

In this review, we considered the role of miRNAs in the cross-talk between tumour and non-tumoral TME cells to promote metastasis. Further understanding of these processes could be useful to develop new treatments for metastatic cancer patients and identify new biomarkers with the ability to improve the management and follow-up strategies for cancer patients.

## 2. Biogenesis and Delivery of miRNAs

**Figure 1:**
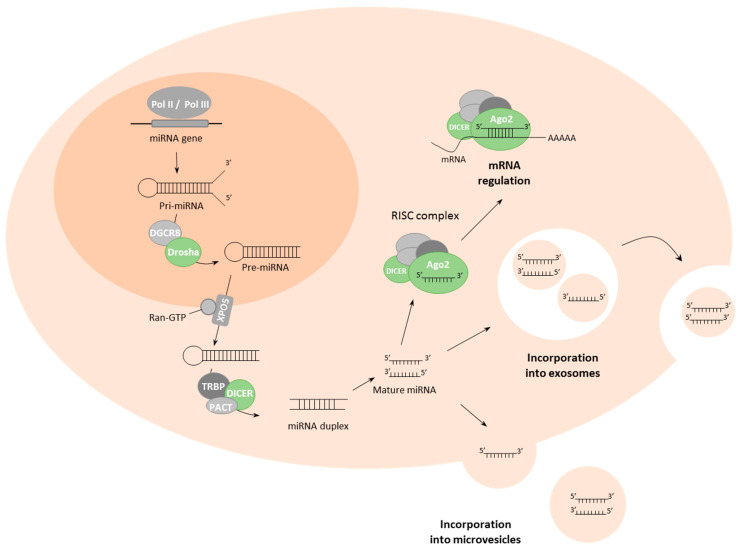
MiRNA biogenesis and its localization. MiRNAs are processed by polymerase II/III to form a pri-miRNA, which is processed by DGCR8 and Drosha in the nucleus. MiRNAs leave the nucleus through interaction with XPO5; in the cytoplasm, miRNAs are processed by a group of proteins, the most important being the RNAse III endonuclease Dicer, resulting in mature miRNAs. These miRNAs can be added to the RISC complex to regulate mRNA expression or can be incorporated into extracellular vesicles (exosomes and microvesicles) to reach other cells in the tumour microenvironment or cells in the premetastatic niche. Pol II / Pol III, polymerase II and III; DGCR8, DiGeorge syndrome critical region 8; XPO5, exportin-5; TRBP, HIV TAR RNA-binding protein; PACT, protein activator of PKR; RISC complex, RNA-induced silencing complex; Ago2, Argonaute 2; miRNA, microRNA; mRNA, messenger RNA.

MiRNA biogenesis starts with the transcription of pri-miRNA sequences from the DNA, with approximately half of miRNAs encoded within intragenic sequences, mainly from introns with the remainder transcribed from intergenic regions and regulated by specific promoter regions [29]. Approximately half of pri-miRNAs encode for multiple miRNAs in a cluster. MiRNA biogenesis can follow either canonical or non-canonical pathways [30]. The canonical miRNA biosynthetic pathway starts with transcription of the pri-miRNA sequence by RNA polymerase II/III; then, RNase-III endonucleases Drosha in concert with the DGCR8 (DiGeorge syndrome critical region 8) cofactor cleave pri-miRNA to form a hairpin pre-miRNA structure [31]. DGCR8 acts to recognize motifs within the pri-miRNA, such as N6-methyladenylated GGAC, while Drosha cleaves pri-miRNA at the base of the structure [31,32]. The resultant pri-miRNA hairpin structure is exported to the cytoplasm by the exportin-5 (XPO5)/RanGTP complex [33], where it is processed by the RNAse III endonuclease Dicer, which removes the terminal loop of the pre-miRNA structure, resulting in a mature miRNA duplex (Figure 1) [34]. The duplex separates into single-strand effector miRNAs which are loaded into the Argonaute (AGO) protein to form the RNA-induced silencing complex (RISC) that regulates expression of target genes through binding of the miRNA to (primarily) the 3′UTR region of mRNA [35,36], although instances exist whereby miRNAs can bind to the 5’UTR region, promoter regions and even the coding sequence [37,38,39]. Gene regulation by miRNAs primarily occurs at the post-transcriptional stage and many mechanisms have been described, the majority of which act negatively although positive regulation has also been described [40].

**Figure 2:**
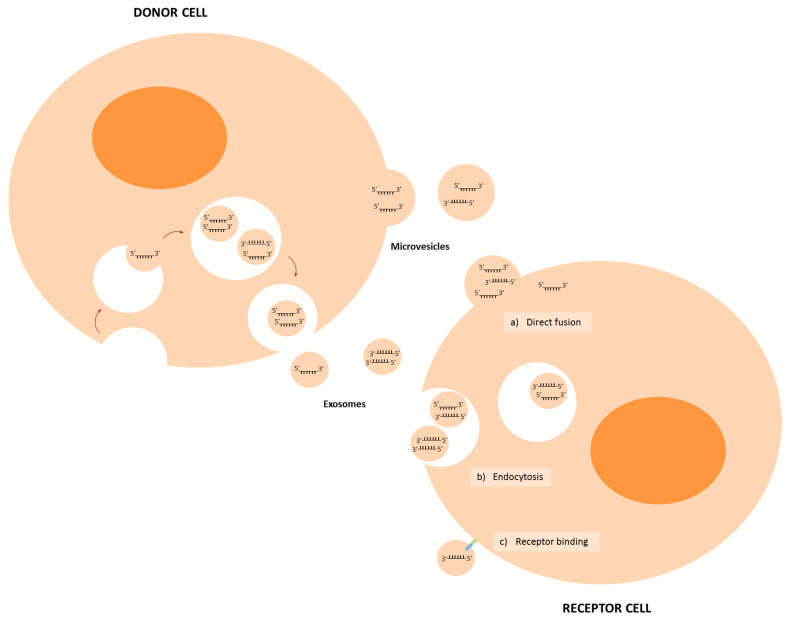
Cross-talk pathways between cells in the tumour microenvironment. It has been described that donor cells secrete miRNAs mainly inside exosomes and microvesicles; these could reach and enter the receptor cell through three different mechanisms: direct fusion, endocytosis and receptor binding. In the recipient cells, these miRNAs perform their function in the case of cancer favouring tumour growth and/or metastasis.

In addition to their role within the cells, miRNAs can act extracellularly leading to great interest in their role as cellular messengers [41]. The most intensely studied form of extracellular miRNAs involved in cell-to-cell communication is extracellular vesicles (EVs), although it should be noted that some controversy remains as to the relative importance of this format with some authors suggesting that most extracellular miRNAs exist in the free form bound to proteins or lipids [42,43]. EVs consist in a lipid bilayer membrane containing lipids, proteins and nucleic acids (including miRNAs) derived from the original cell, which protected their content from enzymatic degradation during transit through the extracellular microenvironment [44]. EVs can be classified mainly into microvesicles (100–1000 nm) and exosomes (50–100 nm) [45]; during the metastatic process, it has been described that they participate in communication between cells and in the preparation of the premetastatic niche [46,47,48]. Extracellular miRNAs are taken up by receptor cells; this internalization can occur by different mechanisms, such as direct membrane fusion, endocytosis and receptor binding, which could trigger a downstream cascade or produce internalization of the vesicle (Figure 2) [49,50]. Several miRNAs have been described to participate in the communication between tumour cells and between tumour cells and tumour stromal cells and regulate metastasis (Table 1).

## 3. MiRNAs in Intercellular Communication in the TME

**Figure 3:**
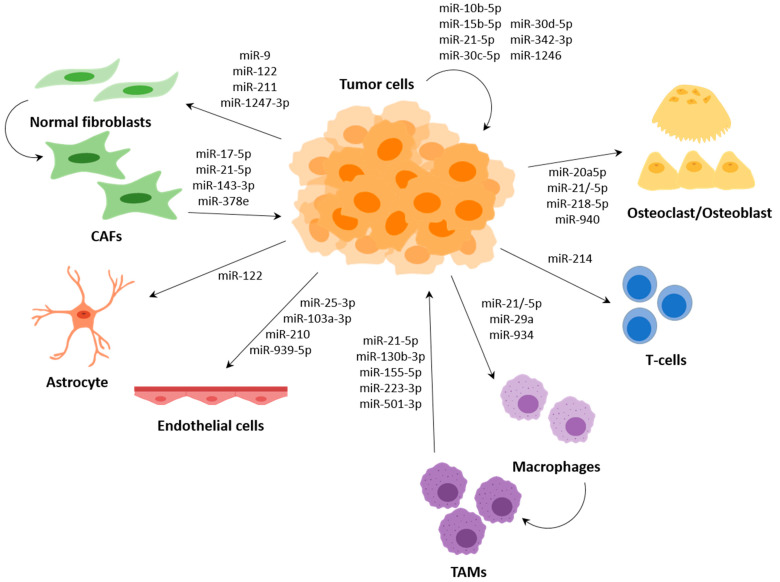
Diagram of the metastasis miRNAs involved in communication between cells in the tumour microenvironment. Tumour cells and cells from the tumour microenvironment, such as fibroblasts, astrocytes, endothelial cells, macrophages, T cells, osteoclasts and osteoblasts, communicate via miRNAs, mainly incorporated into EVs. Tumour cells communicate with each other in the tumour microenvironment to increase its malignancy. Moreover, communication between tumour cells and fibroblasts is mainly related with the switch to the CAF phenotype, while communication between CAFs and tumour cells increase tumour cell mobility and metastasis, similar to what occurs in macrophages where transformation to TAMs is promoted. Cross-talk with astrocytes is related to preparing a metastatic niche in the brain as osteoclasts and osteoblasts which are related with bone metastasis. Finally, communication with endothelial cells is more related with angiogenesis and inflammation, which at the end promote metastasis. EVs, extracellular vesicles; CAFs, cancer-associated fibroblasts; TAMs, tumour-associated macrophages.

Several miRNAs have been described to participate in the communication between tumour cells and cells of the TME, such as fibroblasts, endothelial cells or immune cells, among others; and several of them regulate expression of genes that are involved in the metastasis process (Figure 3).

### 3.1. Surrounding Tumour Cells and Premetastasis Niche Formation

The acidic microenvironment of the TME has been shown to promote the release of EVs and has been associated with tumour progression and metastasis. In glioblastoma, glioma stem cells in acidic microenvironment secrete EVs with MAPK/ERK (mitogen-activated protein kinase/Ras-dependent extracellular signal-regulated kinase)-targeting miRNAs that produce oncogenic reprogramming of the microenvironment, promoting local tumour infiltration [51]. In hepatocellular carcinoma (HCC), exosomal secretion of miR-21-5p and miR-10b-5p induced by acidic microenvironment promote proliferation, migration and invasion of recipient HCC cells [52].

In the TME, cancer stem cells (CSCs) have been found to be important in tumour maintenance and metastasis [53]. It is generally believed that only a subpopulation of tumour cells are able to initiate metastasis, defined as CSCs [54,55]. Moreover, CSCs also exhibit other traits that drive metastasis, including mobility, invasiveness and apoptotic resistance [56,57,58]. In particular, cross-talk between CSCs and tumour cells via miRNAs has been described in clear cell renal cell carcinoma (ccRCC) where exosomal miR-19b-3p secreted by CSCs were demonstrated to be effectively transferred to tumour cells and to induce EMT in those cells via the targeting of PTEN (phosphatase and tensin homolog) [59]. In addition, highly metastatic cells from oral squamous cell carcinoma (OSCC) were observed to secrete miR-342-3p and miR-1246 packaged in exosomes, which were transferred to poorly metastatic cells and shown to promote cell motility and invasive ability of these cells [60].

In distant metastasis, bone is a preferred site for many types of cancer, such as breast and prostate cancer. Metastatic bone lesions are classified as osteoblastic or osteolytic lesions; these are induced by an imbalance between bone formation (osteoblasts) and resorption (osteoclasts) which can be produced by cancer-secreted miRNA in bone microenvironment [61]. Breast cancer is related with osteolytic bone metastasis and some of the secreted miRNAs described with this phenomenon are miR-20a-5p, miR-218 and miR-21 [62,63,64]. High expression of miR-20a-5p has been found in breast tumour cells and also in their exosomes; overexpression in breast tumour cells promotes migration and invasion, while exosomal miR-20a-5p is transferred to bone marrow macrophages and facilitates osteoclastogenesis [62]. Furthermore, in breast cancer, miR-218-5p has been described upregulated in blood samples from patients with bone metastasis; further analysis of this miRNA showed that EV-associated miR-218-5p can downregulate type I collagen expression and deposition by osteoclasts thereby decreasing bone formation and mediating bone niche adaptation, promoting bone metastasis [63]. MiR-21 has also been described to promote differentiation and activation of osteoclasts in breast cancer and lung cancer, reducing both bone density and promoting bone metastasis [64,65]. On the other hand, prostate cancer is related with osteoblastic bone metastasis. Hashimoto et al. described eight highly expressed exosomal miRNAs in prostate cells that correlated with the osteoblastic phenotype including EV-associated miR-940 that was demonstrated to promote osteogenic differentiation of mesenchymal stem cells (MSCs) in bone microenvironment through targeting of ARHGAP1 (Rho GTPase-activating protein 1) and FAM134A (family with sequence similarity 134, member A) [66].

Finally, in the metastatic niche, glucose availability plays an important role in cellular colonization and metastatic formation. In breast cancer, the EV-mediated transfer of miR-122 from tumour cells was shown to reduce glucose uptake by recipient lung fibroblasts and brain astrocytes both in vitro and in vivo via targeting of PKM2 (pyruvate kinase M2) and GLUT1 (glucose transporter 1) resulting in promotion of colonization and metastasis [67].

### 3.2. Cancer-Associated Fibroblasts (CAFs)

Cancer-associated fibroblasts (CAFs) are a group of activated fibroblasts in the TME with high heterogeneity and plasticity that represent a major component of the tumour stroma through production of many of the molecules that make up the extracellular matrix, including cytokines, chemokines and growth factors. CAFs play an important role in tumour progression and metastasis in many cancers, including breast and colorectal cancers, through multiple pathways. CAFs promote survival, growth, invasiveness and angiogenesis of cancer cells by secretion of growth factors, transporting molecules through EVs, or can remodel the extracellular matrix, which is crucial for cancer cell invasiveness [68].

The origin of CAFs is still poorly understood and it has been suggested that tumour cells can promote the transformation of normal fibroblasts into CAFs through EV-associated miRNAs [69]. For example, in HCC, high-metastatic cells have been shown to secrete exosomal miR-1247-3p, which in turn was demonstrated to be taken up by fibroblasts resulting in their activation, and the resultant CAFs secreted proinflammatory cytokines and promoted tumour stemness, EMT, chemoresistance and lung metastasis [70]. In breast cancer, tumour-secreted EV-associated miR-9 was demonstrated to be taken up by normal recipient fibroblasts enhancing the switch to CAF phenotype and increasing cell motility [71]. In melanoma in situ, specific pigment vesicles (melanosomes) were demonstrated to transport miRNAs to fibroblasts resulting in changes to receptor fibroblasts, including increased proliferation, invasion, migration and proinflammatory gene expression, thereby enhancing invasion and formation of the dermal tumour niche [72].

The contrary situation has also been described with CAFs also able to secrete EV-associated miRNAs to recipient tumour cells [73,74]. For example, in breast cancer, CAF exosomes contained high levels of miR-21-5p, miR-378e and miR-143-3p, which could be transferred to recipient tumour cells, which exhibited increased mammosphere formation capacity, EMT marker production and anchorage-independent cell growth [73]. In colorectal cancer (CRC), miR-17-5p was shown to be highly expressed in CAF exosomes that were transferred to tumour cells leading to targeting of RUNX3 (RUNX family transcription factor 3) which was demonstrated to effect the interaction with MYC and subsequent binding to the TGF-β1 (transforming growth factor beta 1) promoter leading to pathway activation and tumour progression [74].

In addition to intercellular communication by miRNAs between tumour cells and fibroblasts, miRNA expression itself within tumour fibroblasts can be affected by their interaction with tumour cells. Mitra et al. found three differentially expressed miRNAs in ovarian CAFs, two of them downregulated (miR-31 and miR-214) and one upregulated (miR-155), compared with normal fibroblasts which were demonstrated to induce the conversion of normal fibroblasts into CAFs [75]. In breast cancer, miR-200s and miR-205 have been described to mediate reprogramming of normal fibroblasts into CAFs and trigger invasion and angiogenesis, respectively [76,77].

### 3.3. Endothelial Cells

During metastasis, the disruption of the endothelial cell tight junctions and recruitment of new blood vessels called angiogenesis is essential for tumour progression [78,79]. Exosomal miR-939-5p in breast cancer and exosomal miR-103a-3p in HCC have been described to be transferred from tumour cells to endothelial cells and directly target vascular endothelial (VE)-cadherin, leading to the destruction of tight junctions thereby facilitating the transendothelial migration of tumour cells by disruption of endothelial junction integrity [80,81]. Moreover, expression of serum miR-103a-3p in HCC patients has been associated with higher metastasis potential [81].

The protein ZO-1 (zonula occludens-1) is a component of the tight junction, which can also be targeted by exosomal miRNAs. For example, miR-25-3p in CRC and miR-105 in breast cancer both target ZO-1 leading to the promotion of vascular permeability and metastasis [82,83]. Exosomal-associated miR-105 in breast cancer targets directly ZO-1 in endothelial cells [83], while regulation of ZO-1 by exosomal miR-25-3p occurs indirectly, as miR-25-3p targets KLF2 and KLF4 (Krüppel-like factors 2 and 4) in endothelial cells, regulating expression of ZO-1, VEGFR2 (vascular endothelial growth factor), occludin and claudin-5 [82]. Moreover, circulating expression of miR-25-3p is significantly higher in CRC patients with metastasis [82], while miR-105 can be detected in circulation at the premetastatic stage [83].

Increased levels of extracellular miR-210, an important regulator of angiogenesis, was secreted by breast cancer metastatic cells and transferred to endothelial cells, which resulted in enhanced angiogenesis due to increased levels of nSMase2 (neutral sphingomyelinase 2) expression [84]. This miRNA was also secreted by hypoxic breast tumour cells to the neighbouring TME, including endothelial cells, where it targeted vascular remodelling target genes, such as ephrin A3 and PTP1B (protein tyrosine phosphatase non-receptor type 1), resulting in promoted angiogenesis [85]. Exosomal miR-210-3p secreted by HCC tumour cells and delivered also in endothelial cells targeting SMAD4 (SMAD family member 4) and STAT6 (signal transducer and activator of transcription 6) resulted in enhanced angiogenesis [86].

### 3.4. Immune System Modulation by miRNAs

Macrophages are the most abundant infiltrative immune cells present in and around tumours and play a critical role in inflammation [87]. Macrophages are known to polarize, depending on different stimuli, to the M1 phenotype with anti-tumour activity or to the M2 phenotype with pro-tumoral activity. Tumour-associated macrophages (TAMs), which are considered to be M2-like, support different aspects of tumour development, including tumour formation, growth and metastasis [88,89].

The premetastatic inflammatory response generated by TAMs leads to tumour growth and metastasis. MiR-21 and miR-29 secreted by lung tumour cells target TLR8 (toll-like receptor 8) within intracellular endosomes leading to induction of NF-κB (nuclear factor kappa-light-chain-enhancer of activated B cells) and NF-κB-mediated secretion of the proinflammatory cytokines TNF-α (tumour necrosis factor alpha) and IL-6 (interleukin-6) [90]. In bladder cancer, macrophages take up exosomal miR-21-5p from tumour cells leading to promotion of M2 polarization and enhanced migration and invasion of tumour cells [91]. Both CRC cell-derived exosomal miR-934 and hypoxic pancreatic cell-derived exosomal miR-301a-3p were demonstrated to activate PI3K/AKT (phosphatidylinositol 3-kinase/protein kinase B) signalling pathway and enhance metastatic capacity of tumour cells through PTEN targeting [92,93]. PTEN also plays an important role in the regulation of T cells, and it has been demonstrated that EV-associated miR-214 from a range of tumour cells including breast cancer, hepatocellular carcinoma, non-small-cell lung cancer (NSCLC) or pancreatic cancer could transfer to T cells leading to downregulation of PTEN and promoting T-reg (regulatory T cells) expansion and IL-10 (interleukin-10) secretion, which in turn promotes tumour growth and enhanced immune suppression in vivo [94].

MicroRNAs have also been shown to be involved in the recruitment of immune cells to the TME, but rather than through the transfer of miRNAs from tumour cells to immune cells, this occurs through the secretion of attractant molecules. For example, both miR-149 in triple-negative breast cancer (TNBC) and miR-148b in HCC have been demonstrated to target colony-stimulating factor-1 (CSF-1) miRNAs [95,96]. In TNBC, downregulation of miR-149 promoted lung metastasis by enhancing CSF1-dependent recruitment and M2 polarization of macrophages, which also correlated with macrophage infiltration and reduced survival in patient samples [95]. Downregulation of miR-148b in HCC patients correlated positively with recurrence, metastasis and poor prognosis. Moreover, in vitro and in vivo metastatic HCC cells showed decreased levels of miR-148b that correlated with increased CSF1, which promoted HCC growth and metastasis through CSF1/CSF1R (colony-stimulating factor-1 receptor)-mediated TAM infiltration [96]. Similarly, miR-561-5p, which directly target chemokine (C–X3–C motif) ligand 1 (CX3CL1), in metastatic HCC downregulated CX3CL1 leading to low infiltration of CX3CR1 (CX3C chemokine receptor 1)-positive NK cells and resulting in promoted tumorigenesis and metastasis [97].

Moreover, it has been described that TAMs in the TME can release extracellular vesicles with miRNAs, and these can be transferred to tumour cells, generally promoting migration, invasion and metastasis. In CRC, exosomal miR-21-5p and miR-155-5p from macrophages directly target the BRG1 coding gene in tumour cells; this gene is a key factor promoting metastasis [98]. Another example is macrophage-derived EVs in gastric cancer (GC), which contain high levels of miR-130b-3p and promote survival, migration, invasion and angiogenesis in GC cells through the modulation of MLL3 (mixed-lineage leukemia protein 3) and GRHL2 (grainyhead-like protein 2 homolog) [99]. MiR-501-3p has been found to be highly expressed in pancreatic ductal adenocarcinoma (PDAC) tissues and TAM-derived exosomes. Exosomal miR-501-3p promotes cancer cell migration and invasion, as well as tumour formation and metastasis in vivo through regulation of TGFBR3 (transforming growth factor beta receptor 3) [100]. Finally, a study detected miR-223-3p in exosomes released by IL-4 (interleukin-4)-activated macrophages; this miRNA has been shown to transfer to breast tumour cells where it regulates invasion through the Mef2c (myocyte enhancer factor 2C)-β-catenin pathway [101].

## 4. Conclusions and Perspectives

The present review summarizes the role of miRNAs in metastasis with a focus on their role in the communication between tumour cells and TME cells. Deregulated miRNAs have been observed in both tumour and TME cells, highlighting the crucial role of miRNAs and tumour microenvironment in cancer progression and metastasis. Many of these miRNAs are secreted through EVs, mostly contained inside exosomes, and target many important cancer-related genes in recipient cells, many of which are related with the metastatic process (Table 1).

MicroRNAs regulate expression of several genes related with metastasis, but at the same time miRNAs could be regulated by competing endogenous RNAs (ceRNAs). Competing endogenous RNAs contain sequences recognized by miRNAs and act as sponges of them, thus modulating gene expression. Competing endogenous RNAs include long non-coding RNAs (lncRNAs) and circular RNAs (circRNAs) [102]. In nasopharyngeal carcinoma, it has been described that overexpression of lncRNA FAM225A which regulates miR-590-3p and miR-1275 [103] and circRNA CRIM1 which regulates miR-422a [104] promote invasion and metastasis, respectively. Gastric cancer patients showed two differentially expressed circRNAs and both act as ceRNAs [105,106]. One of them, circNRIP1, was upregulated and was demonstrated to target the miR-149-5p/AKT1-mTOR axis promoting migration and invasion [105]. In contrast, circCCDC9 was downregulated and was shown to regulate migration and invasion through sponging of the miR-6792-3p/CAV1 axis [106]. Additionally the lncRNA linc00968 was found to be downregulated in lung adenocarcinoma acting as a sponge for miR-9-5p and miR-21-5p, thereby promoting metastasis through regulation of CPEB3 and SMAD7, respectively [107,108].

The discovery of metastasis-related miRNAs secreted by tumour cells or stromal cells in the TME allows us to use them as prognostic biomarkers in different types of cancer. In breast cancer, a 4-miRNA signature in tissue that could predict high or low risk of lymph node metastasis with an area under the curve (AUC) of 0.841 has been described, with poorer overall survival and disease-free survival in the high-risk group [109]. Another example is in prostate cancer, where tissue expression of miR-346 correlated with the Gleason grade, biochemical relapse and higher recurrence risk [110]. Moreover, metastasis-related miRNAs were also found and detected in bodily fluids, such as serum, plasma and urine, among others, which allows the development of non-invasive metastasis biomarkers facilitating patient management [2]. Serum levels of miR-103a-3p and miR-1247-3p in HCC correlated with higher metastatic potential and lung metastasis, respectively [70,81]. Another example of non-invasive biomarkers is serum exosomal miR-301a-3p levels in pancreatic cancer, which correlated with tumour invasion, lymph node metastasis and poorer overall survival of patients [93]. These are just some examples of studies where the role of metastasis-related miRNAs as prognostic cancer biomarkers has been seen, but the field of study of miRNAs as biomarkers has grown in recent years. However, current studies evaluate their biomarker potential using different conservation, extraction and detection protocols, leading to inconsistent results that make difficult their application to clinical practice [111].

On the other hand, deregulated miRNAs in metastasis could be used as a therapeutic approach. There are two main strategies to modulate miRNAs, restoration of downregulated tumour suppressor miRNA (mimics) or inhibition of overexpressed onco-miRNA (antagomiRs) [112,113]. However, efficient delivery of miRNAs / miRNA inhibitors to target tissues is a major challenge in the transition of miRNA therapy to the clinical practice. Several companies are working on miRNA-based therapies and some of them have entered clinical trials, but only a small proportion of them are directed against cancer (Table 2) [114], so more efforts are needed to bring miRNA therapies from the bench to the clinic. The two main delivery approaches used in miRNA modulation are viral- and non-viral-based systems. Viral vectors, such as lentivirus, adenovirus or adeno-associated viruses are efficient delivery methods, but their systemic toxicity and immunogenicity limit their clinical use [115,116]. For this reason, several researchers focused on non-viral delivery systems, such as nanoparticles or exosomes, to deliver miRNAs / miRNA inhibitors; however, these approaches still present a lower efficiency than viral systems [117,118].

As mentioned above, EVs secreted by tumour cells play an important role in promoting metastasis, so it would be tempting to infer that inhibiting EV biogenesis and secretion could be another promising strategy for cancer therapy. It has been shown that sulfisoxazole, an FDA-approved antibiotic, inhibits secretion of small EVs from breast cancer through interference with ETA (endothelin receptor A) and inhibits cancer progression and metastasis as demonstrated in mouse models [122]. Another study screened more than 4000 compounds to target exosomes from cancer cells. These authors found that manumycin A, a natural microbial metabolite, could inhibit exosome biogenesis and secretion in castration-resistant prostate cancer through inhibition of the Ras/Raf/ERK signalling pathway [123]. In addition to targeting EV biogenesis and secretion, EVs could be prevented from reaching their target cells. For example, it has been reported that treatment with EV-specific anti-CD9 or anti-CD63 significantly decreased breast cancer metastasis to the lungs, lymph nodes and thoracic cavity [124]. Ortiz et al. demonstrated that melanoma EVs could downregulate IFNAR1 (type I interferon receptor) and CH25H (cholesterol 25-hydroxylase) in normal cells to facilitate EV uptake and premetastatic niche formation. Upregulation of IFNAR1–CH25H or treatment with reserpine, an antihypertensive drug, limited melanoma EV uptake by normal cells and inhibited tumour progression and reduced lung metastasis [125].

In summary, metastasis-related miRNA plays an important role in cell-to-cell communication, which helps tumour cells to survive, grow and spread to other organs. Its presence in tumour tissue and body fluids gives us the opportunity to use them to our benefit, either as prognostic biomarkers to improve patient management or by developing new therapies to reverse the effect of these miRNAs. Nevertheless, for these applications, it is necessary to develop and establish standardized approaches, conduct more multicentre studies and improve approaches to EV disruption and miRNA-based therapies.

## Figures and Tables

**Table 1:** List of miRNAs associated with metastasis and TME.

miRNA	Cancer	Donor Cells	Receptor Cells	Target	Ref.
miR-9	Breast	Tumour cells	Fibroblasts	E-cadherin	[71]
miR-10b-5p	HCC	Tumour cells	Tumour cells	-	[52]
miR-15b-5p	GBM	Tumour cells	Non-tumour brain cells	MAPK/ERK	[51]
miR-17-5p	CRC	CAFs	Tumour cells	RUNX3	[74]
miR-19b-3p	ccRCC	CSCs	Tumour cells	PTEN	[59]
miR-20a-5p	Breast	Tumour cells	BMMs	SRCIN1	[62]
miR-21	Lung	Tumour cells	Pre-osteoclasts	PTEN	[65]
	Lung	Tumour cells	Macrophages	TLR8	[90]
miR-21-5p	GBM	Tumour cells	Non-tumour brain cells	MAPK/ERK	[51]
	HCC	Tumour cells	Tumour cells	-	[52]
	Breast	Tumour cells	Osteoclasts	PDCD4	[64]
	Breast	CAFs	Tumour cells	-	[73]
	Bladder	Tumour cells	Macrophages	PTEN	[91]
	Colon	TAMs	Tumour cells	BRG1	[98]
miR-25-3p	CRC	Tumour cells	Endothelial cells	KLF2/KLF4	[82]
miR-29a	Lung	Tumour cells	Macrophages	TLR8	[90]
miR-30c-5p	GBM	Tumour cells	Non-tumour brain cells	MAPK/ERK	[51]
miR-30d-5p	GBM	Tumour cells	Non-tumour brain cells	MAPK/ERK	[51]
miR-103a-3p	HCC	Tumour cells	Endothelial cells	VE-Cadherin	[81]
miR-105	Breast	Tumour cells	Endothelial cells	ZO-1	[83]
miR-122	Breast	Tumour cells	Fibroblasts/astrocytes	PKM2/GLUT1	[67]
miR-130b-3p	Gastric	TAMs	Tumour cells	MLL3/GRHL2	[99]
miR-143-3p	Breast	CAFs	Tumour cells	-	[73]
miR-155-5p	Colon	TAMs	Tumour cells	BRG1	[98]
miR-210	Breast	Tumour cells	Endothelial cells	Ephrin A3	[84]
	Breast	Tumour cells	Endothelial cells	Ephrin A3/PTP1B	[85]
miR-210-3p	HCC	Tumour cells	Endothelial cells	SMAD4/STAT6	[86]
miR-211	Melanoma	Tumour cells	Fibroblasts	IGF2R	[72]
miR-214	Lung	Tumour cells	Treg	PTEN	[94]
miR-218-5p	Breast	Tumour cells	Pre-osteoblasts	Col1a1	[63]
miR-223-3p	Breast	TAMs	Tumour cells	Mef2c	[101]
miR-301a-3p	Pancreatic	Tumour cells	Macrophages	PTEN	[93]
miR-342-3p	OSCC	High-metastatic cells	Low-metastatic cells	-	[60]
miR-378e	Breast	CAFs	Tumour cells	-	[73]
miR-501-3p	PDAC	TAMs	Tumour cells	TGDBR3	[100]
miR-934	CRC	Tumour cells	Macrophages	PTEN	[92]
miR-939-5p	Breast	Tumour cells	Endothelial cells	VE-cadherin	[80]
miR-940	Prostate	Tumour cells	MSCs	ARHGAP1 FAM134A	[66]
miR-1246	OSCC	High-metastatic cells	Low-metastatic cells	DENND2D	[60]
miR-1247-3p	HCC	High-metastatic cells	Fibroblasts	B4GALT3	[70]

ccRCC, clear cell renal cell carcinoma; OSCC, oral squamous cell carcinoma; GBM, glioblastoma multiforme; HCC, hepatocellular carcinoma; CRC, colorectal cancer; PDAC, pancreatic ductal adenocarcinoma; CSC, cancer stem cells; BMM, bone marrow macrophages; MSCs, mesenchymal stem cells; CAF, cancer-associated fibroblasts; TAM, tumour-associated macrophages; Ref, reference.

**Table 2:** Clinical trials of miRNA-based therapeutics to treat cancer.

miRNA	Cancer	Product	Type	Phase	Company	Clinical Trial ID	Ref.
miR-10b	GBM	RGLS5579	AntagomiR	Not iniciated	Regulus Therapeutics	-	-
miR-16	Mesothelioma and NSCLC	MesomiR-1	TargomiR	Phase 2	ENGeneIC	NCT02369198	[119]
miR-34	Liver, lymphoma, melanoma, SCLC, MM, RCC, NSCLC	MRX34	Mimic	Withdrawn	Mirna Therapeutics	NCT01829971 NCT02862145	[120]
miR-155	T cell lymphoma	MRG-106	AntagomiR	Phase 2	MiRagen Therapeutics	NCT03713320	[121]

GBM, glioblastoma multiforme; NSCLC, non-small-cell lung cancer; SCLC, small-cell lung cancer; MM, multiple myeloma; RCC, renal cell carcinoma; Ref, reference.

## Data Availability

Not applicable.
